# Supplementary material for: Prediction-error signals in anterior cingulate cortex drive task-switching
Source: Nat Commun. 2024 Aug 17;15:7088. doi: 10.1038/s41467-024-51368-9 (PMC11330528; doi:10.1038/s41467-024-51368-9)
Supplement: Supplementary file 1 — Supplementary Information [file 41467_2024_51368_MOESM1_ESM.pdf]

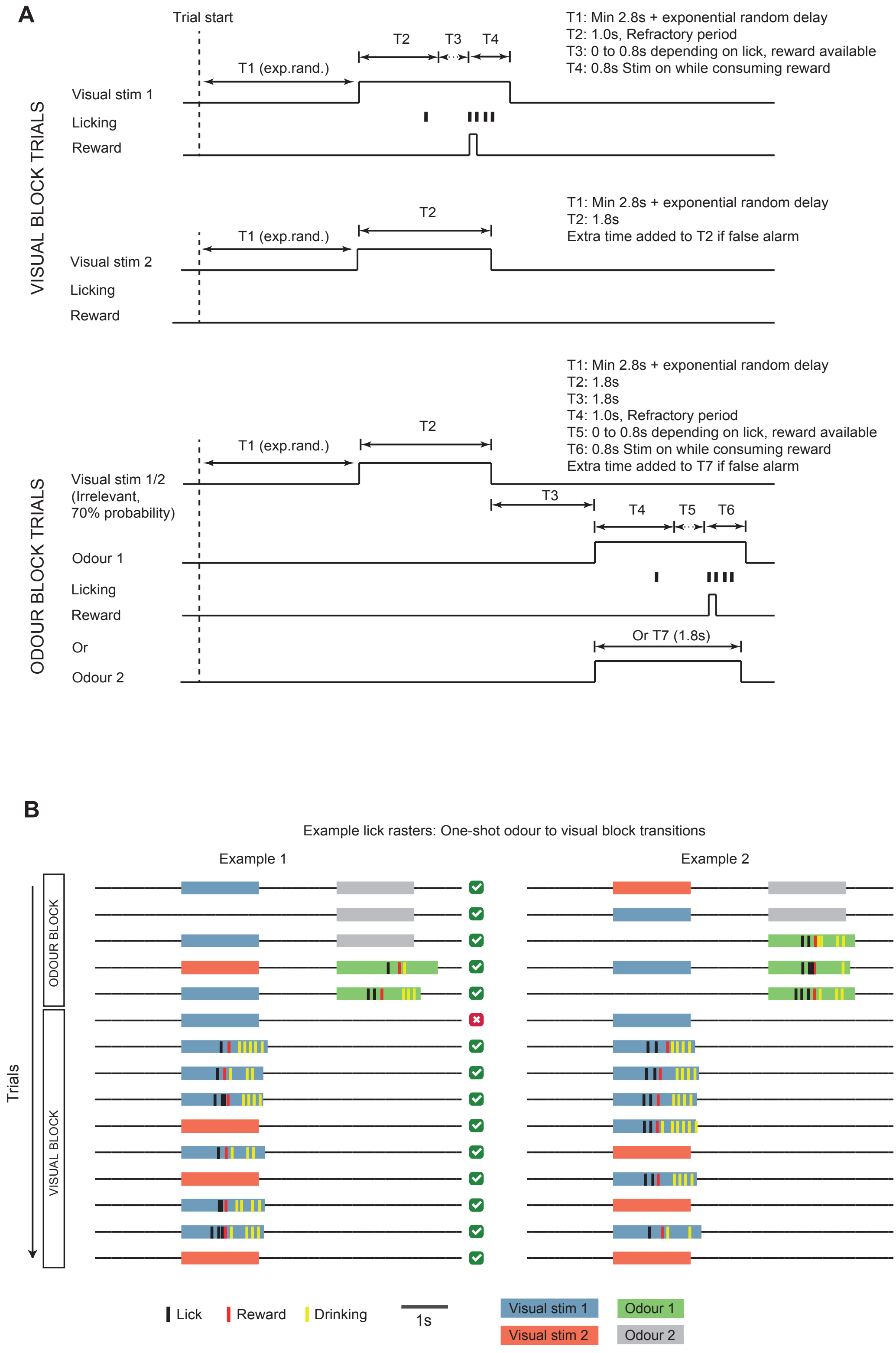

Supplementary Figure 1 | Mice perform rapid task-switching with one-shot block transitions. A. Schematic showing default stimulus durations used in the task, with representative licking and reward valve signals. Top, visual stimulus 1 (rewarded) and 2 (unrewarded) visual block trials. Bottom, example odour 1 (rewarded) or odour 2

(unrewarded) odour block trials. **B**. Example behavioural data from a visual to odour block transition (lick raster) showing stimuli, lick and reward times. In the visual block the mouse discriminates both visual gratings, but switches rules after a single error trial and accurately starts ignoring the same visual stimuli while accurately discriminating odour stimuli.

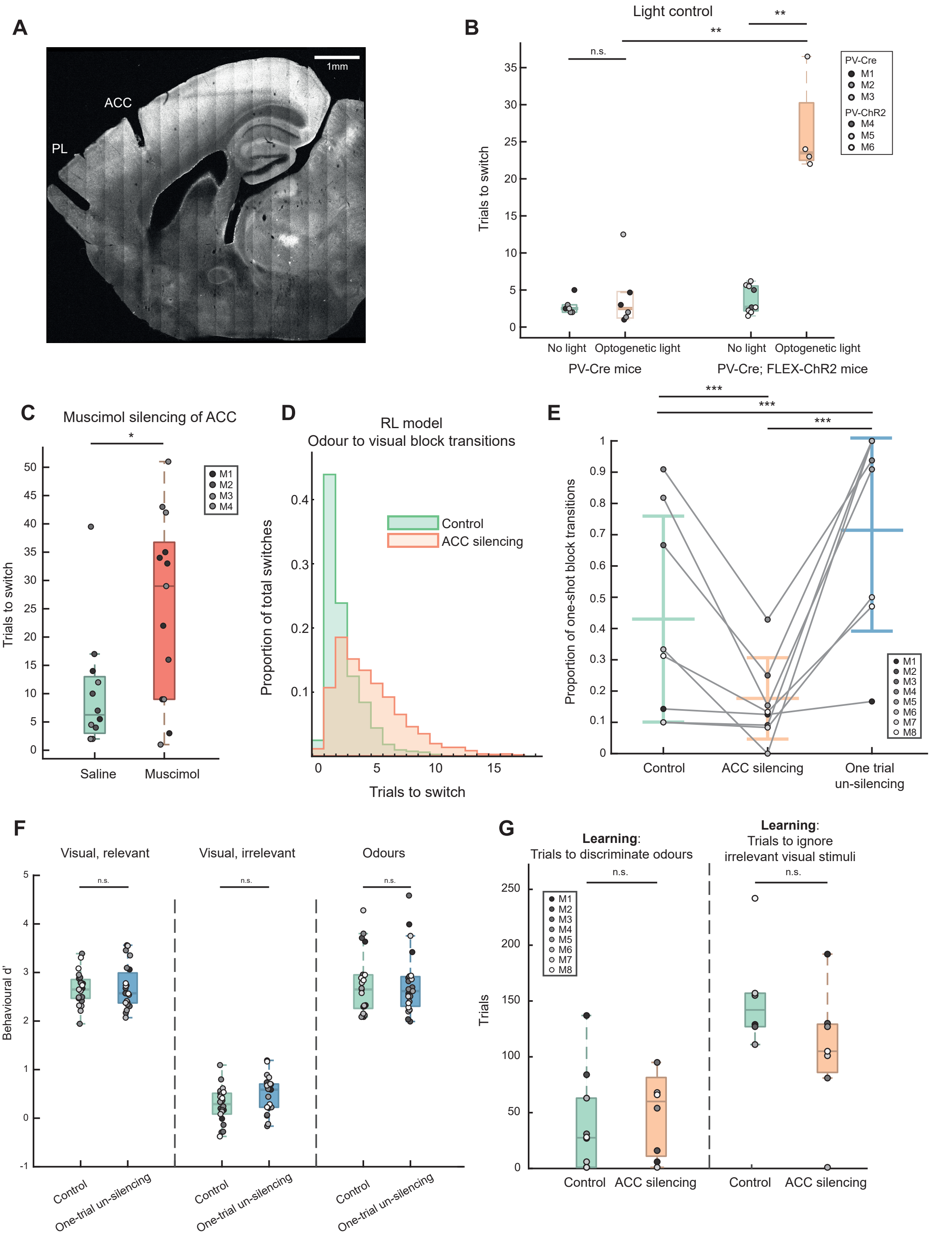

Supplementary Figure 2 | ACC inhibition impairs task switching. A. Sagittal slice from experimental mouse showing relative placements of ACC and PL targeting optic fibre cannulae. **B.** Average number of trials required in a session to switch from an odour block to a visual block, N = 3 PV-Cre (6 sessions per condition) and 3 PV-Cre;FLEX-

Channelrhodopsin2 (9 no light and 4 light sessions) mice, median ± IQR here and below. PV-Cre mice: no light 3 ± 1.21, optogenetic light 3 ± 4.12. PV-Cre;FLEX-ChR2 mice: no light 3 ± 4.56, optogenetic light 23 ± 8.65. Wilcoxon rank sum test comparing PV-Cre mice in light and no-light conditions P = 0.857, comparing PV-Cre and PV-Cre;ChR2 mice in light conditions ** P = 0.0095, comparing PV-Cre;ChR2 mice in light and no light conditions ** P = 0.0028. **C.** Average number of trials per session required to switch from an odour block to a visual block, following bilateral infusions of saline or muscimol into the ACC. Saline infusions, 13 sessions, 6.25 ± 10, muscimol infusions, 14 sessions, 29 ± 27.75, N = 4 mice. Wilcoxon rank-sum comparison P = 0.03. **D.** RL model with belief state fit to the data, with and without ACC silenced. Number of trials taken by the agent to switch from odour to visual blocks increased, leading to fewer one-shot transitions. The model fit the data best when the amplitude of the mismatch signal was reduced by a factor of 0.21. **E.** Proportion of one-shot transitions from odour to visual block. Grey circles represent means for individual mice (N = 8), lines represent means ± STD across mice. Mean ± STD for control 0.43 ± 0.33, continuous ACC silencing 0.18 ± 0.13, one trial un-silencing 0.71 ± 0.32. Chi-squared tests of proportion comparing control and continuous ACC silencing P = 0.0003, control and one trial un-silencing P = 0.0002, continuous ACC silencing and one trial un-silencing P = 0.0002. **F.** Steady-state task discrimination performance after each successful block transition. Behavioural d-primes to visual stimuli in a visual block, median ± IQR: control and one trial un-silencing 2.65 ± 0.39 and 2.57 ± 0.62 respectively, visual stimuli in an odour block 0.29 ± 0.43 and 0.59 ± 0.48, and odour stimuli 2.65 ± 0.69 and 2.62 ± 0.61. Wilcoxon signed-rank test comparing d-primes in control and one trial un-silencing sessions to visual stimuli in visual block P = 0.9757, to visual stimuli in odour block P = 0.0883, to odour stimuli P = 0.9861. **G.** Number of trials required to accurately discriminate odours (>80% accuracy over 30 trials) and ignore irrelevant visual stimuli (100% accuracy over 30 trials) in first task-switching session, for mice with and without continuous optogenetic ACC inhibition (N = 8 and 10 mice respectively), median ± IQR, trials to discriminate odours control 27.5 ± 62 , continuous ACC silencing 60 ± 70.5, trials to ignore irrelevant visual stimuli control 142 ± 30, continuous ACC silencing 105 ± 43.3. Wilcoxon rank-sum comparison of trials to discriminate odours P = 0.35, trials to ignore irrelevant visual stimuli P = 0.11.

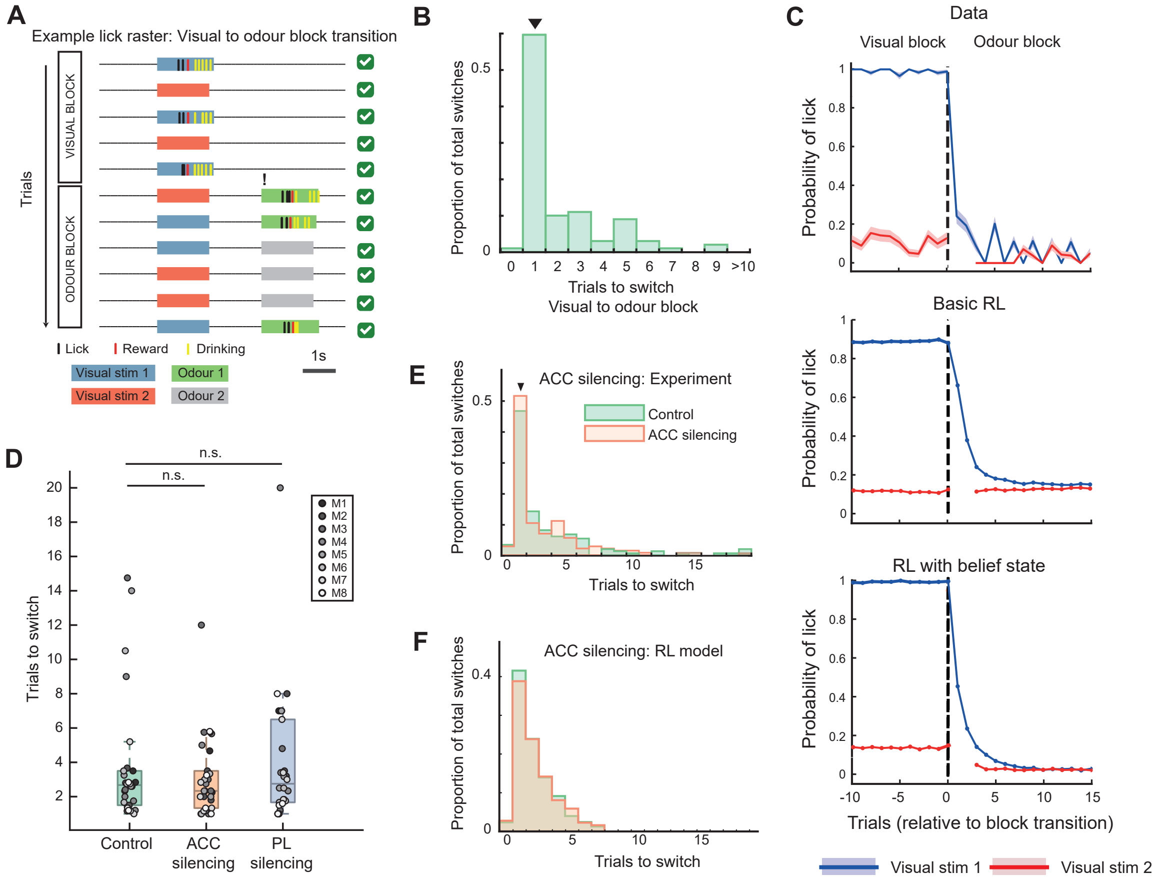

Supplementary Figure 3. Visual to odour block transitions. A. Example behavioural data from a visual to odour block transition (lick raster) showing stimuli, lick, and reward times. In the visual block the mouse discriminates both visual gratings, but switches rules after a single error trial and accurately starts ignoring the same visual stimuli while accurately discriminating odour stimuli. **B.** Histogram showing the number of trials required to switch from visual to odour block, with one-shot transitions indicated by arrowheads. 115 transitions respectively, 17 sessions, 14 mice. **C.** Average probability of licking the reward spout in response to Visual Stimulus 1 (rewarded only in visual blocks) and Visual Stimulus 2 (unrewarded in both blocks), aligned to the block transitions. Data from 115 visual to odour block transitions. Shading indicates SEM. First 3 trials of each block were forced to be Visual Stimulus 1. Middle, basic RL model fit to data. Bottom, RL model with belief state fit to data. **D.** Average number of trials required in a session to switch from a visual block to an odour block, control 2.68 ± 2, continuous ACC silencing 2.33 ± 2.17, continuous PL silencing 2.75 ± 4.83. Wilcoxon signed-rank comparing control and continuous ACC silencing sessions P = 0.81, control and continuous PL silencing sessions P = 0.62, continuous ACC silencing and continuous PL silencing P = 0.91. **E.** Histogram of number of trials taken to switch between blocks during control and continuous ACC silencing sessions. Transitions from visual to odour block, N = 148 and 146 control and ACC silencing transitions respectively. One-shot transitions indicated by arrowhead. **F.** RL model with belief state fit to the data, with and without ACC silenced, as in Supplementary Figure 2D. Number of trials taken by the agent to switch from visual to odour blocks did not change significantly. The model fit the data best when the amplitude of the mismatch signal was reduced by a factor of 0.5.

A

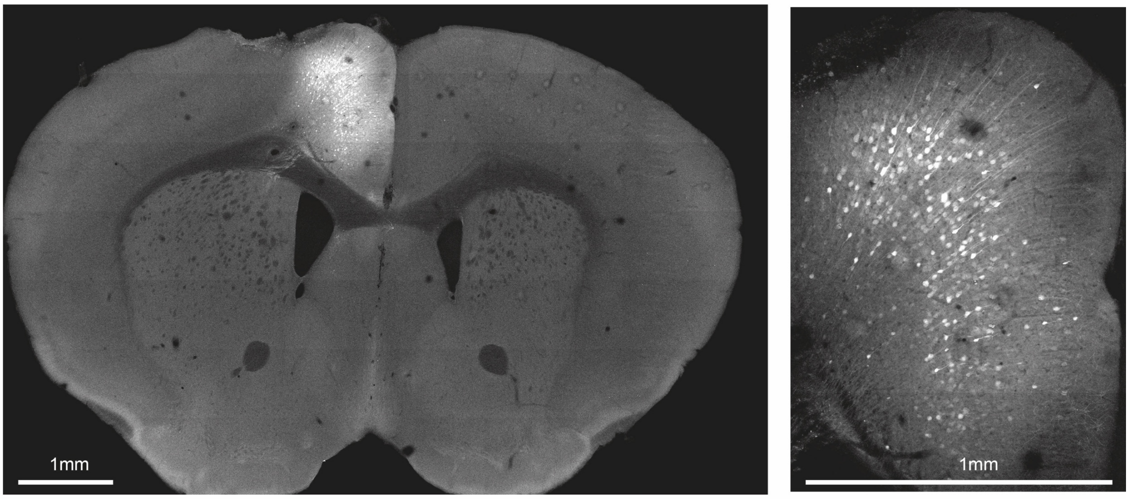

B

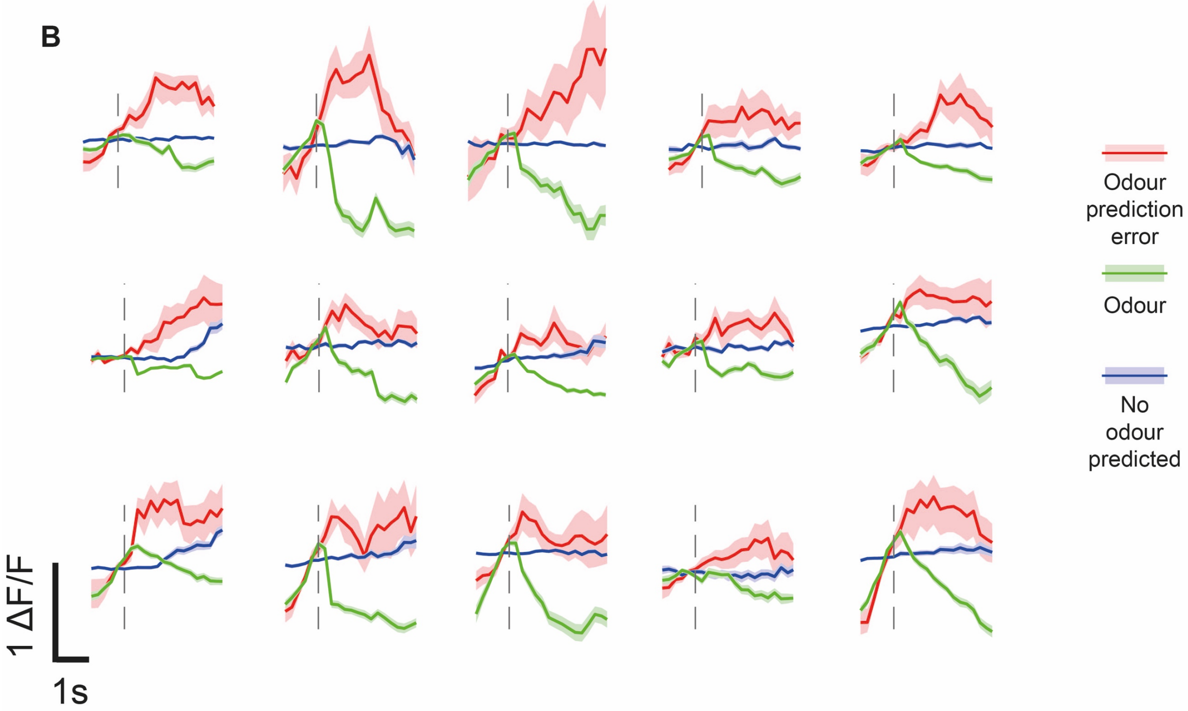

Supplementary Figure 4 | ACC neurons respond to prediction errors. A. Example coronal slice (~+1.2mm anterior relative to bregma) showing GCaMP7f expression relative to the whole brain (left) and within the ACC (right). **B.** Example prediction-error neurons identified from ACC recordings in the 10 imaged mice, showing averaged responses in the odour prediction error, odour, and no odour predicted trials, -1 to 3s relative to real or predicted odour onset.

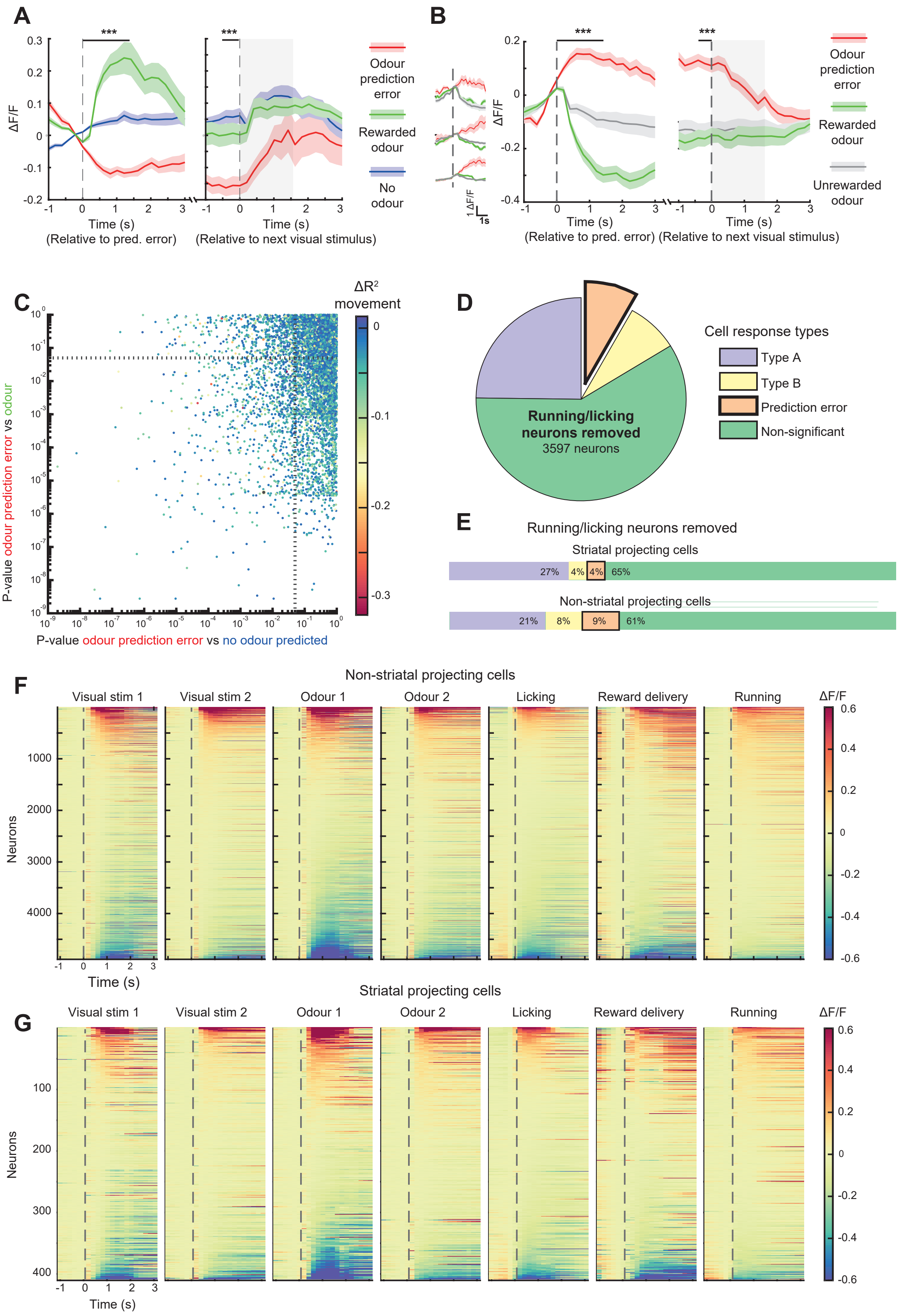

Supplementary Figure 5 | Response properties of ACC neurons. A. Mean response of all negatively responding prediction-error neurons (N = 186 cells) aligned to the odour prediction error event, and to the next visual stimulus onset (grey shading). Response shown to the odour prediction error event (red) and actual odour delivery (green). Wilcoxon signed-rank

test, prediction-error response vs baseline averaged 0 to 1.5 s from prediction error event and -0.5 to 0 s from the next stimulus onset, *** P = 1.61 x 10^{-28}, P = 6.57 x 10^{-17} respectively, prediction error response vs odour response, *** P = 2.97 x 10^{-19}, P = 1.26 x 10^{-6} respectively. **B.** Mean response of all positively responding prediction-error neurons (N = 168 cells) aligned to odour prediction error event, and to the next visual stimulus onsets (grey shading), shown with responses to the odour prediction error event (red) and both the actual rewarded odour (green) and unrewarded odour (grey) delivery. Wilcoxon signed-rank test between the odour prediction error response and unrewarded odour response averaged 0 to 1.5s from prediction error event and -0.5 to 0.s from the next stimulus onset, *** P = 1.69 x 10^{-28}, P = 5.45 x 10^{-15}. **C.** The influence of running and licking on each neuron was inferred by modelling all stimuli, reward delivery, licking and running, and comparing the R^2 with and without the running and licking terms included. The change in R^2 is plotted in colour code for each neuron (N = 6878 cells). Prediction-error neurons had significantly different activity between the prediction error vs odour, and prediction error vs no odour conditions (lines for P < .05 shown on each axis). Neurons with stronger influence of running and licking (more negative ΔR^2) were not enriched in prediction-error neurons. **D.** Proportions of cells as in Figure 4h, after removing cells which were influenced by running (N = 3597 cells). **E.** Proportions of cells as in Figure 4l, after removing cells which were influenced by running (N = 202 and 2437 striatal projecting and non-striatal projecting cells respectively, same colour legend as **D**). **F.** Mean responses of all non-striatal projecting ACC neurons (N = 4888 cells) aligned to task stimuli and behaviour. Activity is aligned to -1s to 3s around task stimulus or behaviour onset and mean baseline (-0.5s to 0s) is subtracted. Each condition is sorted by the averaged activity from 0 to 1s. **G.** As in **F**, but for striatal projecting neurons (N = 421 cells).

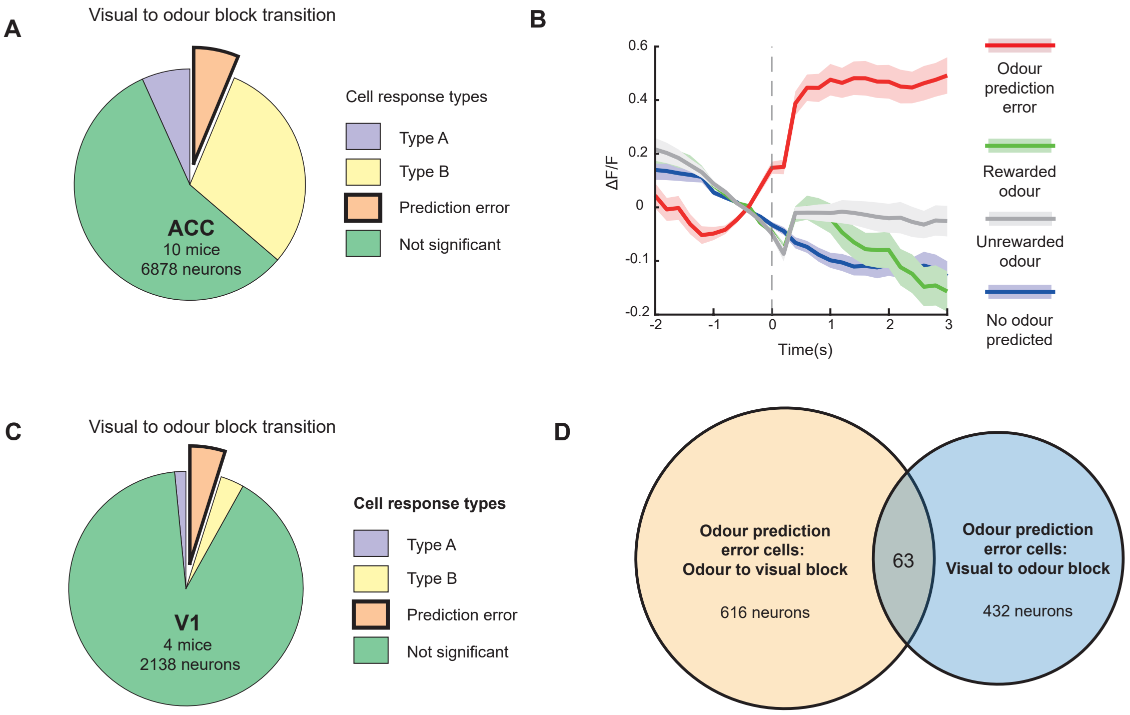

Supplementary Figure 6 | Prediction-error neurons in visual to odour block transitions. **A.** Proportions of cells, as in Figure 4h, for the visual to odour block transition. N = 463 Type A, 6.7%, 2063 Type B, 29.9%, and 432 prediction-error neurons, 6.2%, total 6878 cells, 10 mice. **B.** Mean response of all positive prediction-error neurons (N = 432 cells) aligned to the odour prediction error event (arrival of an unexpected odour). Response shown to the odour prediction error event (red), actual odour delivery of odour 1 (green) and odour 2 (grey), and trials from the visual block where odour is neither predicted nor delivered (blue). **C.** As in **A**, for primary visual cortex (V1). N = 33 Type A, 1.5%, 69 Type B, 3.2%, and 104 prediction-error neurons, 4.8%, total 2138 cells, 4 mice. **D**. Venn diagram showing the overlap between prediction-error neurons in odour to visual block transitions and prediction-error neurons in visual to odour block transitions. The proportion of overlapping cells is significantly higher than chance (bootstrap test, P < 0.01).

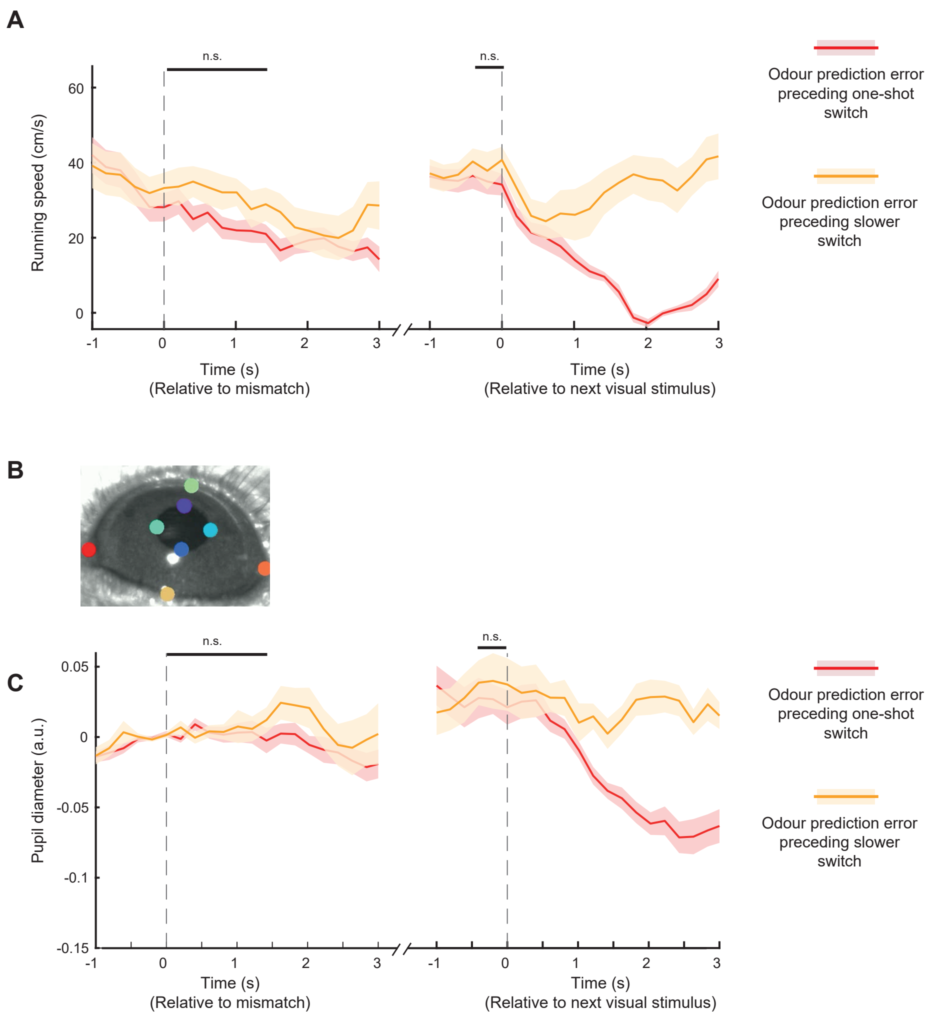

Supplementary Figure 7 | Running and pupil size during one-shot and slower prediction errors. A. Averaged running speed during odour prediction-error trials preceding either one-shot or slower switches, or odour trials. Wilcoxon signed-rank test comparing running speeds in odour prediction-error trials preceding one-shot and slower switches, aligned to expected odour onset 0 to 1.5s P = 0.101, aligned to the next visual stimulus (grey shading) -0.5 to 0s P = 0.413. **B.** Example frame from eye camera recording, with coloured markers used to track eye width/height and pupil width/height using DeepLabCut. **C.** Averaged pupil area during odour prediction-error trials preceding either one-shot or slower switches, or odour trials. Wilcoxon signed-rank test comparing pupil area in odour prediction-error trials preceding one-shot and slower switches, aligned to expected odour onset 0 to 1.5s P = 0.734, aligned to the next visual stimulus (grey shading) -0.5 to 0s P = 0.4258.
