# Supplementary material for: Prediction-error signals in anterior cingulate cortex drive task-switching
Source: Nat Commun. 2024 Aug 17;15:7088. doi: 10.1038/s41467-024-51368-9 (PMC11330528; doi:10.1038/s41467-024-51368-9)
Supplement: Supplementary file 3 — Description of Additional Supplementary Files [file 41467_2024_51368_MOESM3_ESM.pdf]

Description of Additional Supplementary Files

File name: Supplementary Movie 1

Description: Movie showing a one-shot odour to visual block transition. The mouse receives visual stimuli from a monitor, seen in the background, and odour stimuli from an olfactometer delivered through a tube visible on the edge of the frame. Arrival of the odour is indicated by text on screen. The mouse licks the reward spout to trigger a drop of soy milk on correct trials. The video shows the final two trials of an odour bock, where the mouse correctly ignores the visual stimuli (first two trials, visual stim 2 is the irrelevant visual stimulus) and receives an odour after a delay (both trials odour 1), to which it responds by licking the spout and receiving a reward. On the first trial of the visual block, the mouse ignores the visual stimulus (visual stim 1) but does not receive any odour, thus experiencing an odour prediction error. The next trial onwards the mouse switches to applying the rule of the visual block and correctly licks in response to visual stim 1 (next three trials), but not visual stim 2 (next trial). The text above the mouse indicates the rule the mouse is currently holding in mind.
